# Supplementary material for: Connectome dysfunction in patients at clinical high risk for psychosis and modulation by oxytocin
Source: Mol Psychiatry. 2024 Jan 19;29(5):1241–52. doi: 10.1038/s41380-024-02406-x (PMC11189815; doi:10.1038/s41380-024-02406-x)
Supplement: Supplementary file 1 — Supplementary Material [file 41380_2024_2406_MOESM1_ESM.pdf]

Connectome dysfunction in patients at clinical high risk for psychosis and modulation by oxytocin

Cathy Davies & Daniel Martins, Ottavia Dipasquale, Robert A. McCutcheon, Andrea De Micheli, Valentina Ramella-Cravaro, Umberto Provenzani, Grazia Rutigliano, Marco Cappucciati, Dominic Oliver, Steve Williams, Fernando Zelaya, Paul Allen, Silvia Murguia, David Taylor, Sukhi Shergill, Paul Morrison, Philip McGuire, Yannis Paloyelis & Paolo Fusar-Poli

SUPPLEMENTARY MATERIAL

Supplementary Methods

- Participants
- Sample size
- Design, Materials, Procedure
- Nasal Spray Administration
- MRI Acquisition
- Resting-State fMRI Preprocessing and Denoising
- Overlap with Large-Scale Resting-State Networks
- Definitions of Graph Metrics: Supplementary Figure S1

Supplementary Results

- Metric-Level Results: Supplementary Figures S2-S4
- Table S1 - CHR-P vs healthy control group differences in nodal betweenness-centrality, degree and local efficiency as derived from placebo conditions only
- Tikhonov Partial Correlation results: Global Metrics
- Table S2 - Effects of group, treatment and interaction effects on nodal betweenness-centrality, degree and local efficiency (Tikhonov partial correlation)
- Measures of emotional state

References

SUPPLEMENTARY METHODS

Participants

Thirty male, help-seeking CHR-P individuals aged 18-35 were recruited from a specialist early detection service in London, UK (1). A CHR-P status was determined using the Comprehensive Assessment of At-Risk Mental States (CAARMS) 12/2006 criteria (2). Briefly, subjects met one or more of the following subgroup criteria: (a) attenuated psychotic symptoms, (b) brief limited intermittent psychotic symptoms (BLIPS, psychotic episode lasting <1 week, remitting without treatment), or (c) either schizotypal personality disorder or first-degree relative with psychosis (2), all coupled with functional decline. Individuals were excluded if there was a history of previous psychotic disorder (with the exception of BLIPS, some of whom may meet acute and transient psychotic disorder criteria (3)) or manic episode, exposure to antipsychotics, neurological disorder or current substance-use disorder, estimated IQ <70, acute intoxication on the day of scanning, and any contraindications to MRI or intranasal oxytocin or placebo. Seventeen healthy male controls, aged 19-34, were recruited as part of a related study (4). Controls were screened for psychiatric disorders using the Symptom Checklist-90-Revised (5) and Beck Depression Inventory-II (6) questionnaires, were medication free, had no history of drug abuse, tested negative on a urine screening test for recreational drugs, and consumed <28 units of alcohol per week and <5 cigarettes per day. In both studies, subjects were asked to abstain from using recreational drugs for at least 1 week and alcohol for at least 24 hours prior to each session. Urine screening was conducted before each session. The study received Research Ethics Service approval (14/LO/1692 and PNM/13/14-163) and all subjects gave written informed consent.

Sample Size

Several factors were considered when determining sample sizes. As reported in our previous publication (7), at the time of designing our CHR-P study there was no direct effect size (for effects of oxytocin on neuroimaging parameters in CHR-P) from previous studies on which to base our power calculations. However, a meta-analysis of the neurophysiological effects of oxytocin in controls performing social cognition fMRI tasks reported combined effect sizes (d) of 0.82 and 1.56 (large effects) in the amygdala and temporal lobes, respectively (8). A power calculation using G*Power3 indicated that 30 subjects in a within-subject design was sufficient to detect an effect size (dz) of 0.53 (medium effect size), for $\alpha=0.05$ (two-tailed) and $\beta=0.20$ (power of 80%), allowing for potentially smaller effects than reported by the meta-analysis. This was important for the within-subject element of the CHR-P study, given that we examined a range of different neuroimaging/neurophysiological measures, including cerebral perfusion, metabolite concentrations, task-based fMRI and resting state data. We therefore needed

sufficient power to perform reasonable hypothesis testing in each of these domains, and to reduce the possibility that 'negative' findings (i.e. lack of significant effects) would arise due to data insensitivity, which has been highlighted as a critical shortcoming of the wider oxytocin literature (9,10). We thus opted to recruit 30 CHR-P patients to ensure sufficient power in the within-subject aspect, which would also surpass that needed for a between-group analyses when combined with the healthy control data. For the healthy control study, we determined sample size based on our previous studies demonstrating that N=16 healthy controls per group was sufficient to quantify standard nasal spray oxytocin-induced changes in cerebral perfusion in both between-(11) and within-subject (12) designs. We therefore recruited 17 healthy male volunteers. Although the CHR-P and control samples are somewhat unbalanced in size, it is not uncommon to include more patients than controls in MRI studies and this configuration kept the balance between statistical power for the different contrasts and studies, research ethics (having sufficient but not excessive numbers of participants/patients take part) and feasibility.

A final consideration is that the power of a study is not only determined by sample size but also the signal-to-noise ratio of the measures themselves. We attempted to maximise this by using outcome measures derived from data that has theoretically higher signal-to-noise ratio than standard acquisitions. Specifically, we used multi-echo data, which we denoised using a data-driven approach (ME-ICA) and by additionally regressing out the signals from white matter and cerebrospinal fluid. The combination of multi-echo data with this denoising pipeline has been shown to provide better signal-to-noise ratio as compared to single-echo acquisitions or multi-echo acquisitions denoised with other suboptimal approaches (13), i.e. a standard regression of motion parameters and white matter and cerebrospinal fluid signals.

Design, Materials, Procedure

CHR-P sample

For descriptive purposes, we also collected information on medication history, use of alcohol, tobacco and cannabis, and functioning using the Global Functioning (GF) Role and Social scales (14).

Healthy control sample

The healthy control study employed a double-blind, placebo-controlled, triple-dummy, crossover design, where participants took part in 4 experimental sessions spaced 8.90 days apart on average (SD = 5.65, range: 5-28 days). In each session, participants received treatment via three different oxytocin/placebo administration routes, in one of two fixed sequences: either nebuliser/intravenous infusion/standard nasal spray, or standard nasal

spray/intravenous infusion/nebuliser, according to the treatment administration scheme presented in Fig. 8 in (4). In 3 out of 4 sessions only one route of administration contained the active drug; in the fourth session, all routes delivered placebo or saline. Participants were randomly allocated to a treatment order (i.e. a specific plan regarding which route delivered the active drug in each experimental session) that was determined using a Latin square design. Unbeknown to the participants, the first treatment administration method in each session always contained placebo, while intranasal (spray or nebuliser) oxytocin was only delivered with the third treatment administration. This protocol maintained double-blinding while avoiding the potential washing-out of intranasally deposited oxytocin (as might be the case if oxytocin had been administered at the first treatment administration point and placebo at the third administration point). For the current analyses, only the placebo and spray datasets were included, to match the data collected in CHR-P participants.

Justification for combining the CHR-P and healthy control study data

Although the two study designs differed slightly, almost all major aspects were the same across studies. The studies were run in the same facility, using the same MRI scanner, the same acquisition sequences conducted at the same post-dosing timings, the same drug manufacturer/bottles/drug and the same drug administration device/protocol. The key differences are thus that in addition to receiving spray oxytocin/placebo, the healthy controls would have had a saline (placebo) infusion (and thus venous cannulation) and were administered a placebo via nebulizer on the same day as the spray oxytocin and spray placebo data (the data used in this study) were collected. Controls also attended the facility four times in total, rather than two for CHR-P. Although there would be no conceivable active pharmacological effects of these placebos administered intravenously or via nebulizer, it is possible that the placebo effect, or mild stress/pain associated with initial venepuncture or ongoing cannulation, could have an effect on the resting-state data, although the resting state acquisition started at least 1 hour after initial venepuncture. While it would undoubtedly have been advantageous to conduct a single study with both groups undergoing identical procedures, the total number of scans within the present analysis (94) is not trivial for a single-centre pharmaco-MRI study that includes a CHR-P group. Therefore, given that the only alternative is to analyse the within-subject effects of oxytocin vs placebo in CHR-P patients, without an ability to examine these questions against a healthy control comparator, we believe that our findings add important evidence to the literature—with the obvious caveats—which should now be followed up with specifically designed studies. Capitalising on the available evidence in this way is particularly important in the CHR-P field: there is a dearth of evidence supporting the effectiveness of preventative interventions (15) and there are currently no licensed pharmacological treatments to offer to these help-seeking patients (16,17), which

represents a critical unmet clinical need. Experimental medicine studies with potential novel compounds are therefore essential, particularly in terms of providing some initial signal of effects in brain regions/neurophysiological targets of interest, and indeed recent (18,19) (as well as earlier (20)) reviews have recommended exploring oxytocin as a potential novel treatment for these patients. Overall, for these reasons we believe that—in the absence of current alternatives (and notwithstanding the methodological caveats)—combining data from these two studies is important, timely and justified as it enabled us to examine the effects of oxytocin on parameters thought (and shown here) to be altered in CHR-P patients vs controls, and provides the initial evidence of signal of interest that should now be followed up in future optimally-designed studies.

Unique vs overlapping features of the current work compared to previous publication in healthy controls

The healthy control data used in the present study are a subset of data collected and reported in a previous related publication (4). The present analysis is unique from the published triple-dummy crossover study in that we only used data from two of the arms (spray oxytocin, spray placebo), to match the data collected in the CHR-P study. In terms of the analysis, the triple-dummy study was within-subject and set out to examine the effects of different routes of oxytocin administration. Although we have used the resting-state data from those two arms of the triple-dummy study, we have not repeated any of the same analyses of the earlier study, because all of the analyses in the current study include data from CHR-P patients in each contrast performed: main effects of group (collapsed across treatments), main effect of treatment (collapsed across groups), and interaction effects. The CHR-P data is completely novel and has neither been used nor published previously. However, the present work does follow the general steps used in the previous paper (as would be expected in studies of network metrics of multi-echo data), including the choice of graph/network metrics used in the analysis and examination of the dice overlap. However, notable differences include the choice of parcellation scheme and thus number of ROIs, and use of an FSL-based pipeline (this study) rather than CONN (previous study).

Nasal Spray Administration

Participants followed our standard in-house protocol that we have used consistently across all of our studies and is consistent with the guidelines outlined by Guastella et al . Specifically, participants self-administered 10 puffs, each containing 0.1 ml Syntocinon (4 IU) or placebo, one puff every 30 s, alternating between nostrils (hence 40 IU oxytocin in total). Participants blocked the opposite nostril while administering each puff, and each puff was followed by an immediate, brief, sharp, snort.

MRI Acquisition

We aimed to collect resting-state fMRI data at approximately 60 mins post-dosing, in line with previous findings of the spatiotemporal profile of oxytocin-induced changes in cerebral blood flow, which demonstrated sustained effects over a ~20-73 minute period (post-intranasal administration) (11). The resting-state scan was obtained starting at (M±SD) 62.6 ± 3.1 min post-dosing in CHR-P and at 57.01 ± 3.38 min in controls. All scans were conducted on a General Electric Discovery MR750 3 Tesla system (General Electric, Chicago, USA) using a 32-channel head coil. During an 8 min 10 s scan, subjects were asked to lie with their eyes open looking at a centrally-placed fixation cross. Functional data was acquired using a single shot multi-echo echo planar imaging (EPI) sequence (TR = 2500 ms, TE's= 12, 28, 44 and 60 ms, FA= 80°, FOV= 240 mm, matrix= 64 x 64, slice thickness= 3 mm, 32 continuous descending axial slices, resolution = 3.75 × 3.75 × 3 mm, 192 volumes collected for each echo). A 3D high-spatial-resolution Inversion Recovery Spoiled Gradient Echo (IR-SPGR) T1-weighted scan was also acquired (TR/TE/TI = 7328/3024/400 ms; FA= 11°, FOV= 270 mm, slice thickness = 1.2 mm, slice gap = 1.2 mm, matrix = 256 × 256, resolution/voxel size = 1.1 × 1.1 × 1.2 mm).

Resting-State fMRI Preprocessing and Denoising

The multi-echo resting-state fMRI dataset was preprocessed using the AFNI (21) tool meica.py (13,22) and FMRIB Software Library (FSL). Preprocessing steps included volume re-alignment, skull stripping, time-series de-spiking and slice-time correction. Functional data were then optimally combined (OC) by taking a weighted summation of the first three echoes, using an exponential T2 weighting approach (23). While we acquired four echoes, we decided to combine only the first three echoes because unpublished work from our group has shown that the inclusion of the fourth echo can introduce artifacts due to its low signal-to-noise ratio. The OC data were then denoised with the Multi-Echo ICA approach implemented by the tool meica.py (13,22) to remove motion artifacts and other non-BOLD sources of noise. This denoising method has been shown to reduce non-BOLD sources of noise and increase the temporal signal-to-noise ratio more effectively than other standard regression approaches (13,22,24). White matter (WM) and cerebrospinal fluid (CSF) signals were first extracted from each participant's preprocessed datasets using standard eroded WM and CSF masks co-registered to each individual's space (25). Mean WM and CSF signals were then regressed out and a high-pass temporal filter with a cut-off frequency of 0.005 Hz applied.

We used the six rigid-body parameters extracted for each participant using AFNI to calculate the mean frame-wise displacement (FD). One CHR-P subject was removed from further

analyses due to excessive head motion (FD>0.5), while all other participants' motion lied within an acceptable range (FD<0.5) for both sessions. A study-specific template representing the average T1-weighted anatomical image was built using the Advanced Normalization Tools (ANTs) toolbox (26). Finally, each participant's cleaned datasets were co-registered to its corresponding structural scan, then normalised to the study-specific template before warping to standard MNI152 space, with 2mm^3 resampling. The final normalised images were visually inspected to ensure the quality of the preprocessing and the absence of artifacts.

Overlap with Large-Scale Resting-State Networks

To maximise the interpretability of our findings and facilitate comparisons with previous work focusing on large-scale resting-state networks (RSNs) (4,27), we calculated the percentage of overlap between our result maps—which included binary masks of all cortical regions showing differences in nodal metrics (for group, treatment and interaction effects separately)—and the RSNs described in the atlas from Yeo et al (28). As subcortical structures are not covered by the Yeo atlas, these were omitted from our result maps to prevent artificial reduction of the overlap estimate. The Yeo atlas includes a coarse parcellation of seven canonical resting-state networks: the default-mode, dorsal attention, frontoparietal, limbic, somatomotor, visual and ventral attention networks. We created a proxy DKA>Yeo atlas for each of the 7 Yeo RSNs (28) by combining individual DKA regions, allocating each to a single RSN based on the RSN for which each region had the highest number of overlapping vertices based on the confusion matrix from a previous study (29). Overlap was quantified using the Dice-kappa coefficient, which estimates the percentage of voxels of each RSN that overlap with our group/treatment/interaction effect maps. These values provide a qualitative contextualisation of our main findings which the reader can use for quick comparisons with previous literature.

Definitions of Graph Metrics

Supplementary Figure S1. Summary of the graph theory metrics used. Here we summarise the basic concepts (based on (4,30)) of the four graph-theory metrics used in the current study to investigate group (CHR-P vs control), treatment (oxytocin vs placebo) and interaction effects (group x treatment) on the functional connectome. Detailed information on metric calculations can be found in previous publications (30).

Metric	Level	Concept	Schematic example
**Global efficiency**	Global	Average inverse shortest path length. Networks with low average shortest path lengths enable faster and more efficient information transfer. Therefore, those with greater *inverse* shortest path lengths can be thought of as better integrated	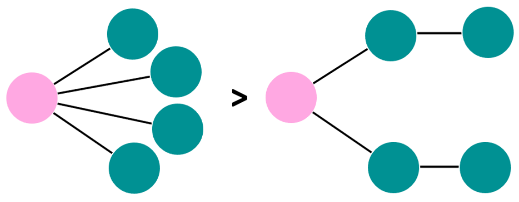
**Local efficiency**	Local	Similar to global efficiency but the metric is computed on the neighbourhood of the given node. Nodes with high local efficiency can be thought of as having greater integration among its neighbours and better fault tolerance if the node is compromised	
**Betweenness-centrality**	Local	Fraction of all shortest paths in the network that pass through a given node. Nodes with high betweenness-centrality can be thought of as “bridging nodes” that connect different parts of the network	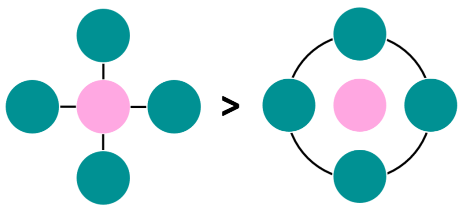
**Node degree**	Local	Number of edges of a node. Nodes with high degree, i.e. with a large number of edges connecting it to other nodes, can be thought of as “hubs”	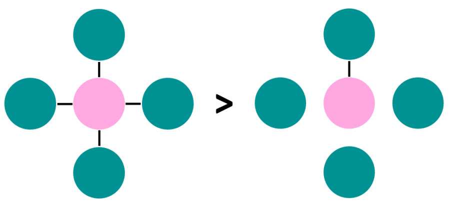

SUPPLEMENTARY RESULTS

Metric-level Results

Results at the level of individual metrics (shown below) were visualised using BrainNet Viewer (31).

Supplementary Figure S2. Overview of group differences in betweenness-centrality, node degree and local efficiency. The pink and green node colours depict lower and greater graph metrics (respectively) in CHR-P relative to healthy controls (HC). The size of each nodal sphere is proportional to the T-statistic of each comparison (axial and sagittal sections scaled separately). ROI abbreviations: cMFG caudal middle frontal gyrus, STG superior temporal gyrus, ITG inferior temporal gyrus, Ling lingual gyrus, lOrbF lateral orbital frontal cortex, ParsT pars triangularis, rMFG rostral middle frontal gyrus, ParsOrb pars orbitalis, PCC pericalcarine cortex, r (suffix) right, l (suffix) left.

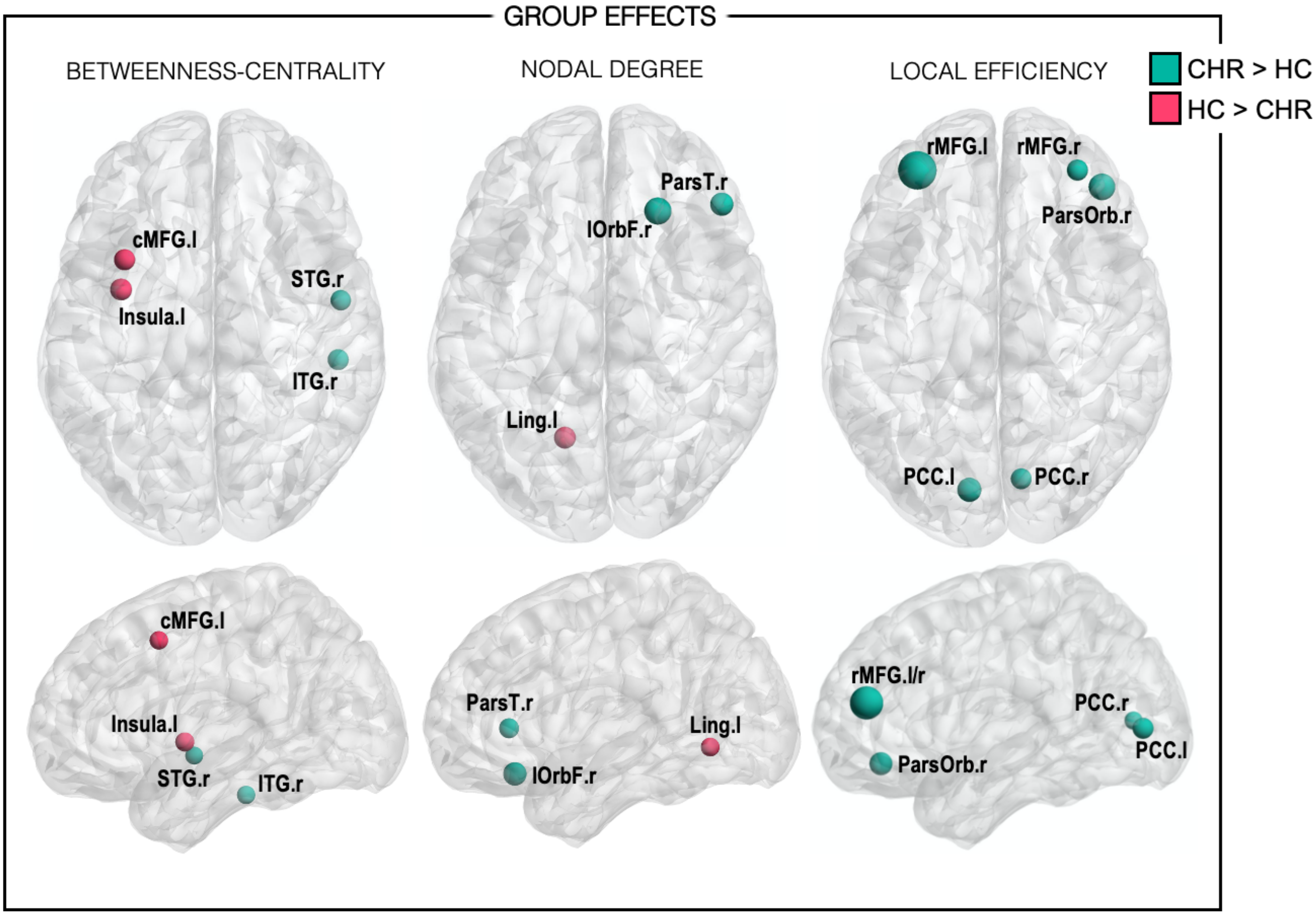

Supplementary Figure S3. Overview of oxytocin (treatment) effects on betweenness-centrality, node degree and local efficiency. The yellow and purple node colours depict increases and decreases (respectively) in graph metrics under oxytocin (OT) relative to placebo (PL). The size of each nodal sphere is proportional to the T-statistic of each comparison (axial and sagittal sections scaled separately). ROI abbreviations: SMG supramarginal gyrus, PrecG precentral gyrus, ParacL paracentral lobule, ParsOp pars opercularis, r (suffix) right, l (suffix) left.

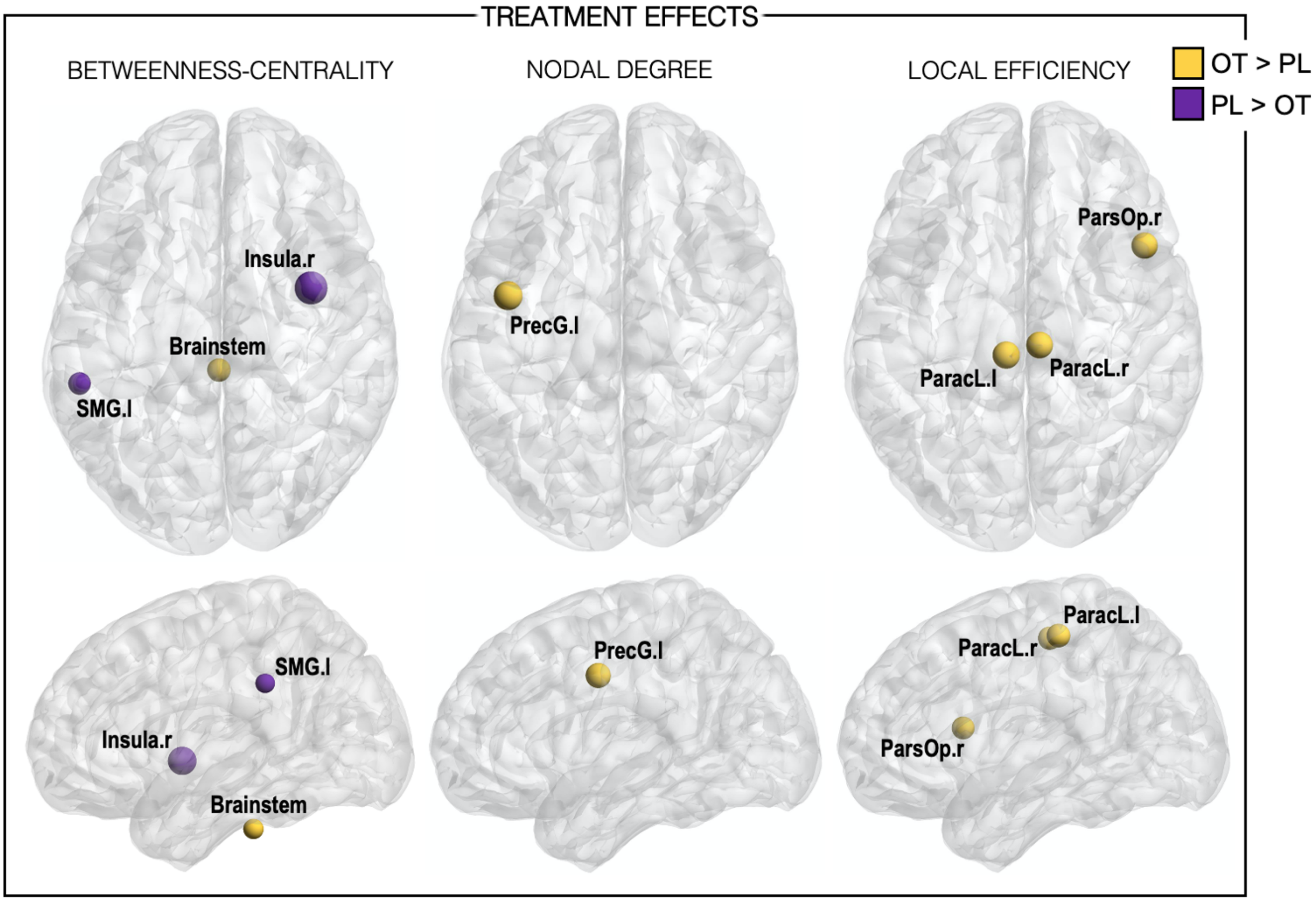

Supplementary Figure S4. Overview of group x treatment interaction effects on betweenness-centrality, node degree and local efficiency. The pink nodes depict where oxytocin (OT) increased (↑) graph metrics in the CHR-P group but decreased (↓) them in healthy controls (HC). The green nodes depict where oxytocin decreased graph metrics in the CHR-P group but increased them in controls. The size of each nodal sphere is proportional to the T-statistic of each comparison (axial and sagittal sections scaled separately). ROI abbreviations: SFG superior frontal gyrus, NAcc nucleus accumbens, Pall pallidum, PrecG precentral gyrus, Thal thalamus, Precun precuneus cortex, EHC entorhinal cortex, TP temporal pole, PCC pericalcarine cortex, r (suffix) right, l (suffix) left.

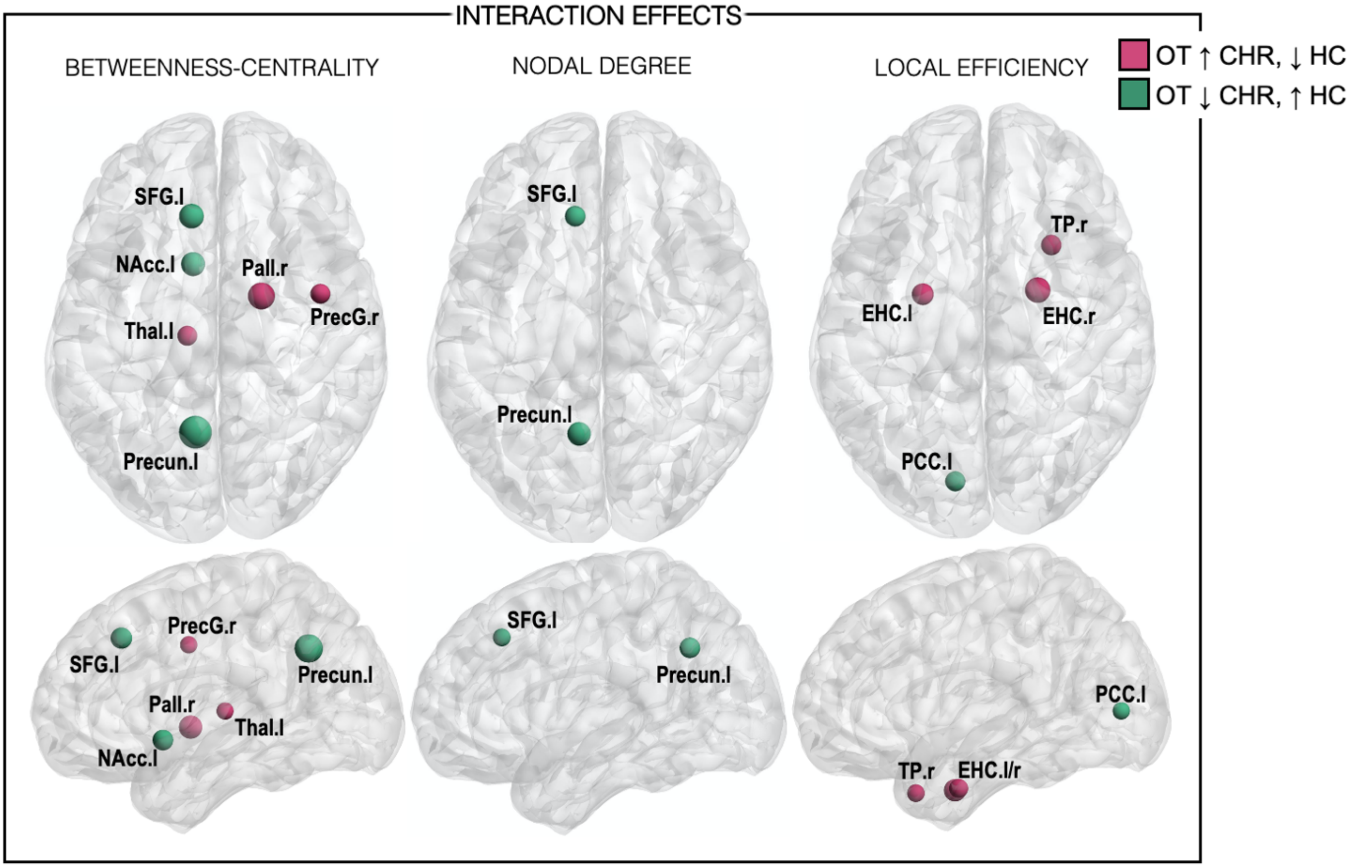

TABLE S1. CHR-P vs healthy control group differences in nodal betweenness-centrality, degree and local efficiency as derived from placebo conditions only.

GROUP DIFFERENCES (PLACEBO CONDITIONS ONLY)			
**Node**	**Direction**	**T statistic**	**P (FDR-corr)**
**Betweenness-Centrality**			
Insula, left	HC > CHR	-2.281	0.028
Thalamus, left	HC > CHR	-2.198	0.034
Pallidum, right	HC > CHR	-2.429	0.019
**Node degree**			
Lateral orbital frontal cortex, right	CHR > HC	2.494	0.017
**Local Efficiency**			
Pericalcarine cortex, left	CHR > HC	3.088	0.004
Rostral middle frontal gyrus, left	CHR > HC	3.066	0.004
Pericalcarine cortex, right	CHR > HC	2.676	0.010

Footnotes: FDR-corr indicates FDR-corrected P-values. OT, oxytocin; PL, placebo; CHR, Clinical High Risk for Psychosis; HC, healthy control.

Tikhonov Partial Correlation Results: Global Metrics

Main Effect of Group (CHR-P vs Healthy Controls)

There were no significant group effects on mean functional connectivity ($t(43) = -0.41$, $p = .69$) nor global efficiency ($t(43) = 0.21$, $p = .84$).

Main Effect of Treatment (Oxytocin vs Placebo)

There were no significant treatment effects on mean functional connectivity ($t(44) = -0.043$, $p = .97$). However, there was a significant treatment effect in terms of global efficiency ($t(44) = -2.11$, $p = .041$), with lower global efficiency in the oxytocin (M+SD = 0.122 + 0.013) relative to the placebo (0.126 + 0.012) condition.

Interaction Effects (Group x Treatment)

There were no significant interaction effects on mean functional connectivity ($t(43) = -0.54$, $p = .59$) nor global efficiency ($t(43) = 0.87$, $p = .39$).

Group Differences (CHR-P vs Healthy Controls – Placebo Conditions Only)

There were no significant group differences in mean functional connectivity ($t(43) = -0.17$, $p = .87$) nor global efficiency ($t(43) = -0.26$, $p = .79$) when comparing the placebo conditions alone.

TABLE S2. Effects of group, treatment and interaction effects on nodal betweenness-centrality, degree and local efficiency (Tikhonov partial correlation)

GROUP EFFECTS			
**Node**	**Direction**	**T statistic**	**P (FDR-corr)**
**Betweenness-Centrality**			
Cuneus, right	CHR > HC	3.391	0.002
Postcentral gyrus, right	CHR > HC	2.255	0.029
Hippocampus, left	HC > CHR	-2.091	0.042
**Node degree**			
Lingual gyrus, right	HC > CHR	-2.088	0.043
Caudate, right	CHR > HC	2.541	0.015
**Local Efficiency**			
Pericalcarine cortex, left	CHR > HC	2.154	0.037

TREATMENT EFFECTS			
**Node**	**Direction**	**T statistic**	**P (FDR-corr)**
**Betweenness-Centrality**			
Inferior parietal cortex, left	PL > OT	-3.197	0.003
Fusiform gyrus right	OT > PL	2.421	0.020
Temporal pole, right	PL > OT	-2.113	0.040
**Node degree**			
Inferior parietal cortex, left	PL > OT	-2.181	0.035
Lingual gyrus, left	OT > PL	2.218	0.032
**Local Efficiency**			
Caudal middle frontal gyrus, left	PL > OT	-2.023	0.049

INTERACTION EFFECTS			
**Node**	**Direction**	**T statistic***	**P (FDR-corr)**
**Betweenness-Centrality**			
Middle temporal gyrus, right	OT > PL in CHR; OT < PL in HC	2.202	0.033
Transverse temporal gyrus, right	OT < PL in CHR; OT > PL in HC	-2.611	0.012
Putamen, left	OT > PL in CHR; OT < PL in HC	2.200	0.033
Accumbens, right	OT < PL in CHR; OT > PL in HC	-2.106	0.041
**Node degree**			
Middle temporal gyrus, right	OT > PL in CHR; OT < PL in HC	2.048	0.047
Transverse temporal gyrus, right	OT < PL in CHR; OT > PL in HC	-4.114	<0.001
**Local Efficiency**			
Pericalcarine cortex, right	OT > PL in CHR; OT < PL in HC	2.580	0.013
Ventral diencephalon, right	OT > PL in CHR; OT < PL in HC	2.607	0.012

GROUP DIFFERENCES (PLACEBO CONDITIONS ONLY)			
**Node**	**Direction**	**T statistic**	**P (FDR-corr)**
**Betweenness-Centrality**			
Middle temporal gyrus, right	HC > CHR	-2.077	0.044
**Node degree**			
Superior parietal cortex, left	HC > CHR	-2.020	0.049
Middle temporal gyrus, right	HC > CHR	-2.219	0.032
Caudate, right	CHR > HC	2.217	0.031
Accumbens, right	CHR > HC	2.277	0.028
**Local Efficiency**			
Caudate, left	CHR > HC	2.105	0.041

Footnotes: FDR-corr indicates FDR-corrected P-values. *Interaction t-test compared difference values (oxytocin minus placebo) between groups. OT, oxytocin; PL, placebo; CHR, Clinical High Risk for Psychosis; HC, healthy control.

Measures of Emotional State

Although it is certainly of interest to understand whether oxytocin has effects on emotional state, it should be noted that our studies were primarily designed (and powered) to examine the neurophysiological effects of oxytocin rather than symptom/psychological effects, which would require significantly larger sample sizes. Nevertheless, for completeness we report here the measures of emotional states collected. Overall, different aspects of emotional state were measured in the two studies, but we did not see any effects of oxytocin vs placebo in either.

In the healthy control data, we assessed participants' levels of alertness (anchors: alert-drowsy) and excitement (anchors: excited-calm) using visual analogue scales (0-100) at 3 different time-points during the scanning session (4,12). As reported in our previous publication, we observed a linear decrease over time in participants' levels of alertness and excitement (main effect of time-interval), but there were no significant main effects of treatment or time-interval × treatment interaction (12). Therefore, these indices did not differ between oxytocin and placebo conditions.

In the CHR-P study, we measured baseline (pre-scan and pre-drug) anxiety scores using the STAI, which occurred approximately 70 mins prior to the MRI scan. We also measured post-scan STAI scores approximately 20 mins after the end of the MRI scan. Missing data were imputed using next-observation-carried-backward as described in our previous publication (32). A repeated-measures ANOVA (within-subject factors treatment [oxytocin, placebo] and time [pre-drug, post-drug]) revealed no significant main effect of condition ($F(1,28) = 2.17$, $p = .15$), a significant main effect of time ($F(1,28) = 9.53$, $p = .005$) (with a decrease in scores from pre-drug to post-scan), and no significant interaction ($F(1,28) = 1.14$, $p = .29$).

REFERENCES

1. Fusar-Poli P, Spencer T, De Micheli A, Curzi V, Nandha S, McGuire P. Outreach and support in South-London (OASIS) 2001—2020: Twenty years of early detection, prognosis and preventive care for young people at risk of psychosis. European Neuropsychopharmacology. 2020;39:111–22.

2. Yung AR, Pan Yuen H, McGorry PD, Phillips LJ, Kelly D, Dell'olio M, et al. Mapping the Onset of Psychosis: The Comprehensive Assessment of At-Risk Mental States. Australian & New Zealand Journal of Psychiatry. 2005 Nov 17;39(11–12):964–71.

3. Fusar-Poli P, Cappucciati M, De Micheli A, Rutigliano G, Bonoldi I, Tognin S, et al. Diagnostic and Prognostic Significance of Brief Limited Intermittent Psychotic Symptoms (BLIPS) in Individuals at Ultra High Risk. Schizophrenia Bulletin. 2017 Jan 4;43(1):48–56.

4. Martins D, Dipasquale O, Paloyelis Y. Oxytocin modulates local topography of human functional connectome in healthy men at rest. Communications Biology. 2021 Dec 15;4(1):68.

5. Ruis C, van den Berg E, van Stralen HE, Huenges Wajer IMC, Biessels GJ, Kappelle LJ, et al. Symptom Checklist 90-Revised in neurological outpatients. Journal of clinical and experimental neuropsychology. 2014;36(2):170–7.

6. Sacco R, Santangelo G, Stamenova S, Bisecco A, Bonavita S, Lavorgna L, et al. Psychometric properties and validity of Beck Depression Inventory II in multiple sclerosis. European journal of neurology. 2016 Apr;23(4):744–50.

7. Davies C, Rutigliano G, De Micheli A, Stone JM, Ramella-Cravaro V, Provenzani U, et al. Neurochemical effects of oxytocin in people at clinical high risk for psychosis. European Neuropsychopharmacology. 2019 May;29(5):601–15.

8. Wigton R, Radua J, Allen P, Averbeck B, Meyer-Lindenberg A, McGuire P, et al. Neurophysiological effects of acute oxytocin administration: systematic review and meta-analysis of placebo-controlled imaging studies. Journal of Psychiatry & Neuroscience. 2015 Jan 1;40(1):E1–22.

9. Quintana DS. Revisiting non-significant effects of intranasal oxytocin using equivalence testing. Psychoneuroendocrinology. 2018;87(August 2017):127–30.

10. Walum H, Waldman ID, Young LJ. Statistical and Methodological Considerations for the Interpretation of Intranasal Oxytocin Studies. Biological Psychiatry. 2015;79(3):251–7.

11. Paloyelis Y, Doyle OM, Zelaya FO, Maltezos S, Williams SC, Fotopoulou A, et al. A Spatiotemporal Profile of In Vivo Cerebral Blood Flow Changes Following Intranasal Oxytocin in Humans. Biological Psychiatry. 2016;79(8):693–705.

12. Martins DA, Mazibuko N, Zelaya F, Vasilakopoulou S, Loveridge J, Oates A, et al. Effects of route of administration on oxytocin-induced changes in regional cerebral blood flow in humans. Nature Communications. 2020;11(1):1–16.

13. Dipasquale O, Sethi A, Lagan MM, Baglio F, Baselli G, Kundu P, et al. Comparing resting state fMRI de-noising approaches using multi-and single-echo acquisitions. PLoS ONE. 2017;12(3):1–25.

14. Cornblatt BA, Auther AM, Niendam T, Smith CW, Zinberg J, Bearden CE, et al. Preliminary findings for two new measures of social and role functioning in the prodromal phase of schizophrenia. Schizophrenia Bulletin. 2007;33(3):688–702.

15. McGorry PD, Mei C, Amminger GP, Yuen HP, Kerr M, Spark J, et al. A Sequential Adaptive Intervention Strategy Targeting Remission and Functional Recovery in Young People at Ultrahigh Risk of Psychosis: The Staged Treatment in Early Psychosis (STEP) Sequential Multiple Assignment Randomized Trial. JAMA Psychiatry [Internet]. 2023 Jun 28 [cited 2023 Jul 2]; Available from: https://doi.org/10.1001/jamapsychiatry.2023.1947

16. Davies C, Cipriani A, Ioannidis JPA, Radua J, Stahl D, Provenzani U, et al. Lack of evidence to favor specific preventive interventions in psychosis: a network meta-analysis. World Psychiatry. 2018 Jun;17(2):196–209.

17. Davies C, Radua J, Cipriani A, Stahl D, Provenzani U, McGuire P, et al. Efficacy and Acceptability of Interventions for Attenuated Positive Psychotic Symptoms in Individuals at Clinical High Risk of Psychosis: A Network Meta-Analysis. Frontiers in Psychiatry. 2018 Jun 12;9(JUN):1–17.

18. Bargiota SI, Papakonstantinou AV, Christodoulou NG. Oxytocin as a treatment for high-risk psychosis or early stages of psychosis: a mini review. Frontiers in Psychiatry [Internet]. 2023 [cited 2023 Nov 11];14. Available from: https://www.frontiersin.org/articles/10.3389/fpsyt.2023.1232776

19. Fusar-Poli P, Davies C, Solmi M, Brondino N, De Micheli A, Kotlicka-Antczak M, et al. Preventive Treatments for Psychosis: Umbrella Review (Just the Evidence). Frontiers in Psychiatry [Internet]. 2019 Dec 11;10(December). Available from: https://www.frontiersin.org/article/10.3389/fpsyt.2019.00764/full

20. Millan MJ, Andrieux A, Bartzokis G, Cadenhead K, Dazzan P, Fusar-Poli P, et al. Altering the course of schizophrenia: progress and perspectives. Nature Reviews Drug Discovery. 2016 Jul 4;15(7):485–515.

21. Cox RW. AFNI: Software for Analysis and Visualization of Functional Magnetic Resonance Neuroimages. Computers and Biomedical Research. 1996 Jun;29(3):162–73.

22. Kundu P, Brenowitz ND, Voon V, Worbe Y, Vértes PE, Inati SJ, et al. Integrated strategy for improving functional connectivity mapping using multiecho fMRI. Proceedings of the National Academy of Sciences of the United States of America. 2013;110(40):16187–92.

23. Posse S, Wiese S, Gembris D, Mathiak K, Kessler C, Grosse-Ruyken ML, et al. Enhancement of BOLD-contrast sensitivity by single-shot multi-echo functional MR imaging. Magnetic Resonance in Medicine. 1999;42(1):87–97.

24. Kundu P, Benson BE, Baldwin KL, Rosen D, Luh WM, Bandettini PA, et al. Robust resting state fMRI processing for studies on typical brain development based on multi-echo EPI acquisition. Brain Imaging and Behavior. 2015 Mar 16;9(1):56–73.

25. Collins DL, Holmes CJ, Peters TM, Evans AC. Automatic 3-D model-based neuroanatomical segmentation. Human Brain Mapping. 1995;3(3):190–208.

26. Avants BB, Tustison NJ, Song G, Cook PA, Klein A, Gee JC. A reproducible evaluation of ANTs similarity metric performance in brain image registration. NeuroImage. 2011 Feb;54(3):2033–44.

27. Xin F, Zhou F, Zhou X, Ma X, Geng Y, Zhao W, et al. Oxytocin Modulates the Intrinsic Dynamics Between Attention-Related Large-Scale Networks. Cerebral Cortex. 2018;1–13.

28. Yeo BTT, Krienen FM, Sepulcre J, Sabuncu MR, Lashkari D, Hollinshead M, et al. The organization of the human cerebral cortex estimated by intrinsic functional connectivity. Journal of Neurophysiology. 2011;106(3):1125–65.

29. Alexander-Bloch AF, Shou H, Liu S, Satterthwaite TD, Glahn DC, Shinohara RT, et al. On testing for spatial correspondence between maps of human brain structure and function. NeuroImage. 2018;178(February):540–51.

30. Rubinov M, Sporns O. Complex network measures of brain connectivity: Uses and interpretations. NeuroImage. 2010;52(3):1059–69.

31. Xia M, Wang J, He Y. BrainNet Viewer: A Network Visualization Tool for Human Brain Connectomics. PLoS ONE. 2013;8(7).

32. Davies C, Paloyelis Y, Rutigliano G, Cappucciati M, De Micheli A, Ramella-Cravaro V, et al. Oxytocin modulates hippocampal perfusion in people at clinical high risk for psychosis. Neuropsychopharmacology. 2019 Jun 9;44(7):1300–9.
